# Flagg’s Lateral Cavity Plates

**Published:** 1850-10

**Authors:** J. F. B. Flagg

**Affiliations:** Surgeon Dentist


### FLAGG'S LATERAL CAVITY PLATES.

Messrs. Jones, White & Co.,—*Gentlemen:* The operation for adjusting an entire upper set of teeth to the mouth, is one of great delicacy, and often presents difficulties the most experienced in our art are not altogether prepared to meet. I have reference to those cases that depend entirely upon atmospheric pressure for their adhesion.

The purpose of this communication is to recommend to the profession a plan which I have recently introduced in my practic e,ith the most satisfactory results; and the purpose of the invention is the more perfectly to secure to the upper jaw artificial teeth, when recourse is had to atmospheric pressure; to prevent the rocking or canting of such teeth, when antagonized by the under teeth in mastication, and to restore the upper jaw to its original fullness, so desirable to retain a natural tone of voice.

I am aware that "cavity suction plates" have been more or less used for many years, of various construction, with different degrees of success; and that one has more recently obtained letters patent, under the name of "central cavity plates," for the greater adhesion to the roof of the mouth; but these have required that much metallic substance should be carried over the entire bony palate; conflicting with the sense of taste, and having its *chamber* so located as to cause a protuberance in the mouth, entirely at variance with all anatomical formation; inducing changes from the natural tone of voice, difficulty of articulation, and other serious complaints from many who have resorted to them.

The nature of my invention consists in so forming the plate, upon which the artificial teeth are secured, as to perfectly fit the jaw in all its parts, as at present ordinarily practiced, except in that portion of the dental ridge immediately behind, and in line with the grinding or molar teeth. At this point I recommend that the plate be made sufficiently depressed to have no bearing upon the jaw, thus forming *lateral cavities* or chambers which, when exhausted of the air by suction, secures the whole plate firmly in its proper position. This depression of the plate, also, restores to the jaw that fullness which it had lost by absorption, consequent upon the extraction of teeth.

The alteration which I make in the plate, is accomplished in the following manner: After obtaining an accurate impression of the jaw, in wax, I cut out a portion of the wax along the line of the grinding teeth upon each side of the mould, about one inch in length by three-eighths in width, and one-tenth in depth, of an oval and cup-like form; taking care not to warp or otherwise alter the general character of the mould. Into this wax mould I cast my plaster of Paris; this plaster cast, when sufficiently *set* or hardened, I remove from the wax, and trim with a knife suitable to prepare the necessary metallic casts for striking up the plate. Should the plate not fit the jaw perfectly from this impression, I recommend that a similar plate be made of *sheet lead;* adjust this lead plate to the jaw, *taking care not to derange the suction cavities;* and, by placing this lead carefully in the former wax mould, cast the plaster once more into it and proceed as before.

I remain, gentlemen, truly yours,

J. F. B. Flagg, M. D., *Surgeon Dentist.*

[*Dental News Letter.*

